# Accuracy and Effectiveness of AI-Powered Systems in Patient Counseling, Education, and Management in Optometry and Related Eye-Care Settings: A Systematic Review

**DOI:** 10.3390/healthcare14152270

**Published:** 2026-07-24

**Authors:** Manal M. Alharbi, Emtenan M. Alharbi, Zainab Ali Al-Hakmani, Amr A. Arafat, Melaf Alotaibi, Raneem Alatawi, Hanan Asiri, Amani Aljurayyad

**Affiliations:** 1 Department of Optometry, College of Applied Medical Sciences, King Saud University, Riyadh 11451, Saudi Arabia; zainabah@squ.edu.om (Z.A.A.-H.); amaniopto@gmail.com (A.A.); 2 Clinical Pharmacy Department, College of Pharmacy, King Saud University, Riyadh 11451, Saudi Arabia; 3 Research Department, Prince Sultan Cardiac Center, Riyadh 12611, Saudi Arabia; amr.arafat@med.tanta.edu.eg (A.A.A.); hananasiri.sa@gmail.com (H.A.); 4 Research and Innovation Institute, Ministry of Defense Health Services, Riyadh 12354, Saudi Arabia; 5 College of Medicine, Imam Mohammad Ibn Saud Islamic University, Riyadh 11564, Saudi Arabia; melafalotaibi25@gmail.com; 6 College of Medicine, King Saud University, Riyadh 11451, Saudi Arabia; falraneem@gmail.com

**Keywords:** artificial intelligence, optometry, patient counseling, patient education, ophthalmology, large language models, ChatGPT, systematic review

## Abstract

**Highlights:**

**What are the main findings?**

- This systematic review synthesized 39 eligible studies evaluating AI-supported patient counseling, education, communication, referral/follow-up, and management in optometry and related eye-care settings.
- Patient-facing LLMs and chatbot-based systems were prominent, particularly ChatGPT, Gemini/Bard, ChatGPT-4o, and disease-specific eye-care agents.

**What are the implications of the main findings?**

- Accuracy and readability were commonly evaluated, but patient-centered outcomes such as trust, satisfaction, usability, adherence, safety, and real-world clinical impact remained inconsistently reported.
- Current evidence supports AI as a supervised clinical adjunct rather than a replacement for optometrists, ophthalmologists, or clinician-led counseling.

**Abstract:**

**Background/Objectives:** Artificial intelligence (AI), including large language models, chatbots, machine-learning systems, and hybrid tools, is increasingly used to support patient-facing eye-care communication. This systematic review evaluated the accuracy and effectiveness of AI-powered systems used for patient counseling, education, communication, referral/follow-up, and management support in optometry and related eye-care settings. **Methods:** A systematic review was conducted according to a predefined protocol and PRISMA 2020 reporting principles. Searches covered studies published from January 2020 to 31 May 2026 in PubMed, Embase, Web of Science, Cochrane Library, and IEEE Xplore. Eligible studies were original primary studies evaluating AI-supported tools for patient-facing counseling, education, question answering, treatment or medication guidance, triage, referral, follow-up, screening linked to management, or clinical decision support. Methodological quality was appraised using relevant JBI critical appraisal tools. **Results:** Thirty-nine studies were included in the qualitative synthesis. JBI appraisal indicated heterogeneous study designs and reporting quality, with common limitations related to simulated prompts or AI-output evaluations, variable comparators, and inconsistent outcome reporting. Evidence was dominated by patient-facing large language models and chatbot-based tools used for patient education, question answering, readability improvement, glaucoma and myopia counseling, diabetic retinopathy referral/follow-up, cataract education, oculoplastic and retinal-condition questions, and multilingual educational support. Study designs, AI models, prompting approaches, comparators, clinical topics, and outcome definitions varied widely, supporting narrative synthesis rather than quantitative pooling. **Conclusions:** AI-powered systems show potential as supervised adjunctive tools for eye-care counseling, education, and management-related communication. However, evidence remains heterogeneous and dependent on simulated prompts, AI-generated outputs, and model-based evaluations. Future research should prioritize standardized evaluation, real-patient validation, safety monitoring, readability control, and patient-centered outcomes before routine implementation in optometry and broader eye-care practice across diverse clinical settings.

## 1. Introduction

Artificial intelligence is increasingly integrated into healthcare, with applications ranging from diagnostic support to patient education, communication, and long-term disease management [1,2,3,4,5]. In optometry and related eye-care practice, patient counseling is essential because many eye conditions require ongoing monitoring, treatment adherence, lifestyle education, symptom recognition, and timely referral when warning signs appear [3,4]. Recent eye-care studies in this area have examined AI-supported referral/follow-up and management-support workflows [6,7,8,9,10,11,12,13,14,15,16,17,18], other patient-facing communication applications [19,20,21,22,23,24,25,26,27,28,29,30,31,32,33,34,35,36,37], and recent patient education and question-answering studies [38,39,40,41,42,43,44]. AI-powered tools may assist by generating explanations, answering common questions, producing patient handouts, simplifying medical terminology, and reinforcing information delivered during clinical encounters [38,39,40,41,42,43,44].

Early eye-care AI research demonstrated strong performance for image-based disease screening, particularly diabetic retinopathy detection [1,2]. Although this diagnostic literature established the technical feasibility of AI in eye care, it does not directly answer whether AI-generated patient-facing information is accurate, readable, safe, and clinically appropriate for counseling [1,2,3,4]. Patient-facing AI requires a different standard of evaluation because outputs must be understandable to patients and must not delay or replace professional care [38,39,40,41,42,43,44,45,46,47,48,49,50].

Recent large-language-model studies have expanded from diagnostic or examination-style tasks toward patient education, online question answering, glaucoma counseling, pediatric ophthalmology education, multilingual materials, and real patient questions [38,39,40,41,42,43,44,45,46,47,48,49,50]. These studies suggest that AI tools may generate useful information, but their performance varies by model, prompt, language, clinical topic, comparator, and scoring method [38,39,40,41,42,43,44]. Safety, readability, and patient-centered outcomes remain major concerns [38,39,40,41,42,43,44,45,46,47,48,49,50].

Although previous systematic and scoping reviews have examined broad uses of large language models and artificial intelligence in ophthalmology, most have focused on general ophthalmic applications, diagnostic or educational use cases, or broad LLM performance rather than the specific patient-facing pathway addressed here. The present review therefore provides a distinct synthesis of original primary studies evaluating AI-supported counseling, education, communication, referral/follow-up, and management-related support in optometry and related eye-care settings.

This systematic review focuses on AI-powered systems used for patient counseling, education, communication, referral/follow-up, and management support in optometry and related eye-care settings [38,39,40,41,42,43,44,45,46,47,48,49,50]. Because optometric practice overlaps with broader ophthalmic patient education, ophthalmology studies were included when the AI application was patient-facing and addressed counseling, education, question answering, referral/follow-up, or management support rather than diagnostic image classification alone [45,46,47,48,49,50].

The review question was: What is the accuracy and effectiveness of AI-powered systems used to support patient-facing counseling, education, communication, referral/follow-up, and management-related tasks in optometry and related eye-care settings? Accordingly, this systematic review aimed to synthesize original primary evidence on these patient-facing AI applications and to evaluate their reported accuracy, clinical quality, readability, safety, and patient-centered or implementation outcomes, when available.

### Clinical Significance

The clinical significance of this topic lies in the potential use of AI as a supplementary counseling and education tool [38,39,40,41,42,43,44]. If accurate, readable, and appropriately supervised, AI-generated responses may help reinforce information provided during clinical visits, answer common patient questions, support follow-up instructions, and improve access to patient education materials [38,39,40,41,42,43,44]. At the same time, patient-facing AI may also introduce risk if outputs are incomplete, overly reassuring, difficult to understand, or not aligned with referral urgency [45,46,47,48,49,50]. For this reason, a review focused specifically on patient-facing communication, rather than diagnostic performance alone, is clinically relevant for optometrists, ophthalmologists, and teams considering AI-assisted education or follow-up support.

## 2. Materials and Methods

### 2.1. Study Design and Reporting Framework

This study was conducted as a systematic review using a predefined protocol. Reporting was guided by PRISMA 2020 principles [51], and methodological appraisal was guided by relevant Joanna Briggs Institute (JBI) critical appraisal tools and evidence-synthesis guidance [52]. The review protocol was prospectively registered in PROSPERO (registration number: CRD420251234880). Eligibility criteria, information sources, screening procedures, extraction fields, outcomes, and synthesis methods were defined before the review was conducted. The PRISMA 2020 checklist and complete search strategy are provided in Supplementary File S1.

Protocol amendments were recorded in PROSPERO. These updates mainly refined the title, citation and author details, rationale, domain terms, review objectives, eligibility framework, outcome wording, quality-appraisal approach, search date range, and synthesis plan. The main methodological updates included extending the search date to 31 May 2026, aligning the methodological appraisal approach with JBI tools, and using narrative synthesis rather than meta-analysis because the included studies were highly heterogeneous in AI models, prompts, comparators, clinical tasks, rating scales, and outcome definitions. These updates were made to align the registered record with the final patient-facing scope and eligible evidence base, and they did not change the core focus of the review on patient-facing AI applications in optometry and related eye-care settings.

### 2.2. Information Sources and Search Strategy

The literature search covered studies published from January 2020 to 31 May 2026. Information sources included PubMed, Embase, Web of Science, Cochrane Library, and IEEE Xplore. The search strategy was developed from the eligibility framework using three structured concept blocks: (i) eye-care population or context terms, including optometry, ophthalmology, ocular disease, and related eye-care settings; (ii) AI-intervention terms, including artificial intelligence, machine learning, deep learning, large language models, and chatbots; and (iii) patient-facing function or outcome terms, including counseling, education, communication, question answering, referral, follow-up, management, readability, and understandability. Search terms were adapted to each database interface using appropriate keywords, controlled vocabulary, and Boolean combinations where applicable. Search reporting was aligned with PRISMA-S principles for documenting database-specific search strategies [53]. The complete search strategy is provided in Supplementary File S1. The May 2026 update was treated as an updated search to capture newly eligible studies, and the final 39-study synthesis reflects all records retained after full-text eligibility reassessment and search updating.

### 2.3. Eligibility Criteria

Eligible studies included original primary studies evaluating AI-supported tools in optometry or ophthalmology for patient counseling, patient education, healthcare communication, frequently asked questions, treatment or medication guidance, triage, referral, follow-up, screening linked to patient management, or patient-facing clinical decision support. Eligible AI systems included LLMs, chatbots, machine-learning tools, deep-learning systems, rule-based systems, and hybrid systems. Randomized trials, observational studies, cross-sectional studies, pilot studies, quasi-experimental studies, case studies, case series, and comparative evaluation studies were considered when they reported relevant patient-facing or management-related outcomes.

Reviews, editorials, letters, response articles, survey/review-style papers without primary outcome data, non-eye-care AI studies, examination/training-only studies, and purely technical image classification, segmentation, or prediction studies without patient-facing counseling, education, referral, follow-up, or management outcomes were excluded.

No restriction was applied to the language of AI outputs or patient-facing materials. Studies evaluating non-English or multilingual patient-facing materials were eligible when sufficient information was available in English, or could be reliably interpreted, to assess eligibility and extract the relevant methods, outcomes, and results. Studies were not included if language barriers prevented reliable assessment of eligibility or extraction of outcome data.

Referral, triage, screening, or management tools were included only when the AI application directly supported patient-facing communication, referral, follow-up, or management decisions. Tools used only for clinician-facing technical prediction, segmentation, or classification without patient-facing pathway relevance were excluded.

Studies based on real patient questions or interactions, simulated patient prompts or vignettes, patient education materials, and expert-only AI-output evaluations were eligible when they met the patient-facing or management-related criteria. The eligibility framework is summarized in Table 1.

### 2.4. Study Selection, Data Extraction, and Methodological Quality Appraisal

Records were managed using Covidence systematic review software (Veritas Health Innovation, Melbourne, Australia; available at www.covidence.org). Titles, abstracts, and full texts were assessed against the eligibility criteria by two reviewers independently, with disagreements resolved through discussion or consultation with a third reviewer when consensus was not reached. Data extraction and JBI methodological appraisal were also completed using the same two-reviewer and consensus approach. A formal inter-rater agreement statistic, such as Cohen’s kappa, was not calculated because all discrepancies were resolved by consensus and the review did not use a single quantitative eligibility score.

Data were extracted using a standardized, predefined extraction form implemented as a structured, wide-format spreadsheet with an extraction dictionary. Extracted variables included study identification, publication year, country, study design, setting, participant or evaluation type, AI model name and type, comparator, patient-facing function, reported outcomes, exact outcome values where available, and source traceability. When a study evaluated multiple AI systems, each model was captured separately for model-family summaries, while the study itself remained counted once in the final synthesis.

Methodological quality was appraised using the relevant JBI critical appraisal tool for each study design. Appraisal outcomes were not used as exclusion criteria; instead, they informed the narrative interpretation of confidence in the evidence. Studies with randomized, real-patient, implementation, or clearly reported comparative designs were interpreted as providing stronger support for applied conclusions, whereas studies based on brief reports, simulated prompts, AI-output-only evaluations, unclear sampling, or limited comparator reporting were interpreted more cautiously. Common appraisal concerns included heterogeneous study designs, limited real-patient validation, reliance on simulated prompts or AI-output evaluations, and inconsistent reporting of comparators, prompts, model versions, and patient-centered outcomes. Accordingly, output-level findings on accuracy, readability, or appropriateness were not interpreted as direct evidence of patient understanding, adherence, safety, or real-world clinical effectiveness unless these outcomes were specifically measured.

### 2.5. Outcomes

The primary outcome was the accuracy or clinical quality of AI-generated counseling or educational information, including correctness, completeness, appropriateness, safety, or alignment with clinician/expert judgment, professional guidelines, society educational materials, or other reference standards. Secondary outcomes included readability, reliability, efficiency, comprehensiveness, understandability, usefulness, satisfaction, trust, usability, actionability, safety, potential harm, adherence, and other patient-centered outcomes when reported.

### 2.6. Statistical Analysis and Synthesis

A descriptive synthesis was performed using the final eligible study set (*n* = 39). Studies were grouped by primary AI model or system category, primary patient-facing function, clinical topic, comparator type, reported outcome domain, and JBI appraisal concern level. Categorical variables were summarized descriptively, with 39 used as the denominator when study-level proportions were reported. Outcome-reporting frequencies and model-family summaries were based on the final 39-study dataset. Performance outcomes were extracted exactly as reported by each study.

Because the included studies differed substantially in AI model type, prompt structure, comparator, clinical topic, rating scale, and outcome measurement, meta-analysis or quantitative pooling was not appropriate. Findings were therefore synthesized narratively using tables and figures. Formal certainty-of-evidence rating using the GRADE approach was not undertaken because the heterogeneity in study designs, AI models, comparators, outcome definitions, and reference standards, together with the predominance of simulated-prompt and AI-output evaluations rather than comparative patient outcomes, precluded the outcome-specific evidence syntheses required for GRADE. Certainty was therefore considered narratively alongside the JBI appraisal.

**Figure 1:**
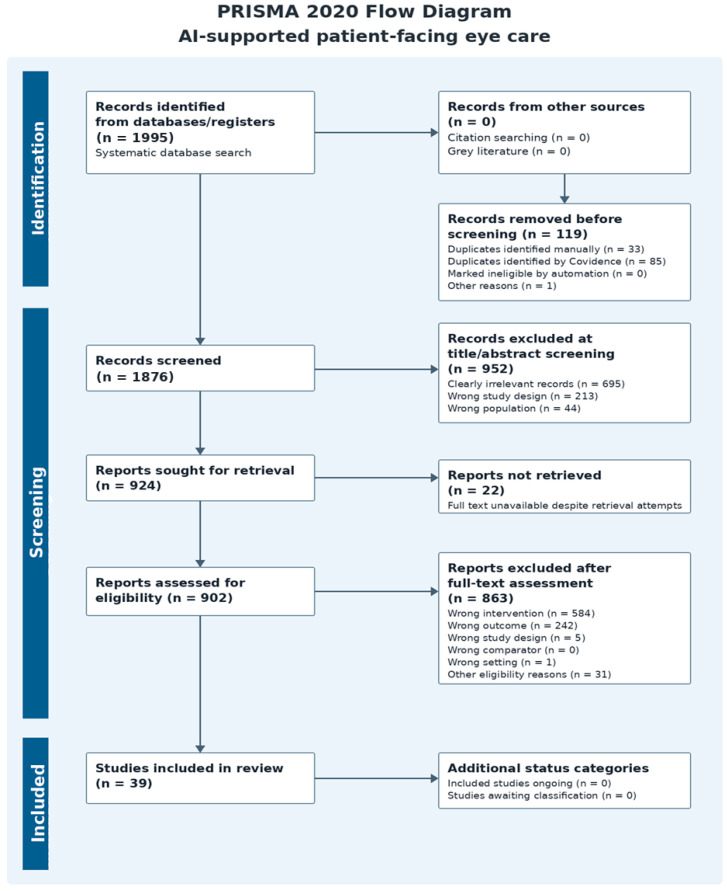
PRISMA flow diagram of study selection for the systematic review of AI-supported patient-facing education, counseling, communication, and management in optometry and ophthalmology.

The study-selection process followed the PRISMA stages of identification, screening, retrieval, eligibility assessment, and inclusion. Eligibility criteria and screening procedures are described in Section 2.3 and Section 2.4, whereas the final study-selection counts are reported in Section 3 and summarized in Figure 1.

## 3. Results

### 3.1. Study Selection

A total of 1995 records were identified from bibliographic databases. Before screening, 119 records were removed, including 33 duplicates identified manually, 85 duplicates identified by Covidence, and one record removed for other reasons. No trial registries were searched; all identified records came from bibliographic database searches.

After removal of these records, 1876 records were screened. At title and abstract screening, 952 records were excluded: clearly irrelevant records (*n* = 695), wrong study design (*n* = 213), and wrong population (*n* = 44). A total of 924 reports were sought for retrieval, and 22 reports were not retrieved because the full text was unavailable despite retrieval attempts. Of 902 reports assessed for eligibility, 863 were excluded after full-text assessment because of wrong intervention (*n* = 584), wrong outcome (*n* = 242), wrong study design (*n* = 5), wrong comparator (*n* = 0), wrong setting (*n* = 1), or other eligibility reasons (*n* = 31).

Thirty-nine studies were included in the final qualitative synthesis. The study-selection process is summarized in Figure 1 using the PRISMA stages of identification, screening, retrieval, eligibility assessment, and inclusion. The PRISMA flow diagram was generated using Covidence, and the narrative text reports the final selection counts used for the 39-study synthesis. The 39 included studies correspond to the studies cited in the main reference list and listed in Supplementary Table S1.

### 3.2. Characteristics of the Final Included Studies

The final synthesis included 39 studies and centered on patient-facing AI applications in optometry and ophthalmology, including patient education, question answering, counseling support, patient handout generation, readability improvement, referral/follow-up support, and management-related communication. Many included studies evaluated LLMs or chatbot-based systems, while others examined AI-enabled triage, referral, or management-support tools linked to patient care pathways. This final set reflects the updated search and final eligibility reassessment rather than a preliminary Covidence export.

To avoid duplication with the reference list, detailed study identifiers and DOI/stable identifier information are retained in Supplementary Table S1, while the main text summarizes the evidence domains, AI model categories, patient-facing functions, outcome domains, and methodological appraisal findings.

**Figure 2:**
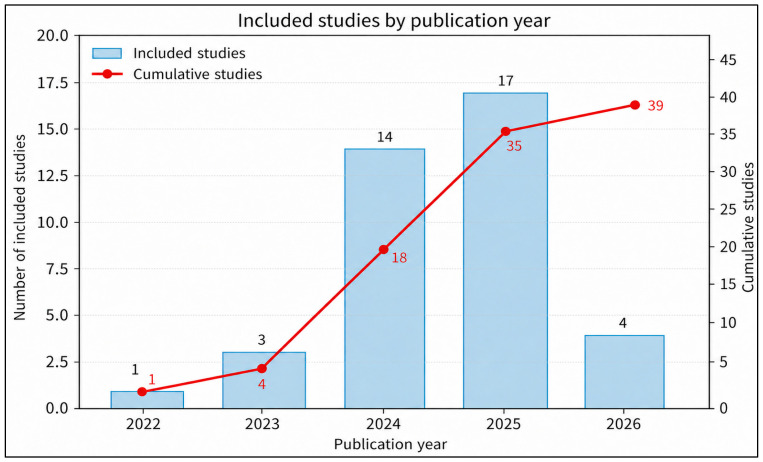
Distribution of included studies by publication year and cumulative study count in the final 39-study synthesis.

The evidence base expanded mainly after 2023, reflecting rapid growth in LLM-based eye-care communication and patient-education research. The studies included a mixture of online LLM-output evaluations, simulated patient questions, patient education materials, real patient questions, randomized or comparative evaluations, and referral/follow-up or management-support studies. Because designs and reporting standards varied, results were synthesized descriptively rather than pooled statistically. The publication-year distribution of the included studies is shown in Figure 2.

The included studies were distributed as follows: one study in 2022, three studies in 2023, 14 studies in 2024, 17 studies in 2025, and four studies in 2026. The 2026 count reflects studies identified up to 31 May 2026 and does not represent a full calendar year.

To strengthen the narrative synthesis, the included studies were grouped by patient-facing application and AI model type. The main application groups included patient education and readability, question answering and counseling, referral/follow-up and management support, and multilingual or accessibility-focused communication. The main model groups included general-purpose LLMs or chatbots, AI screening/referral/follow-up workflows, and disease-specific or multimodal eye-care agents. These domains and examples from the included studies are summarized in Table 2.

### 3.3. AI Models and Patient-Facing Functions

**Figure 3:**
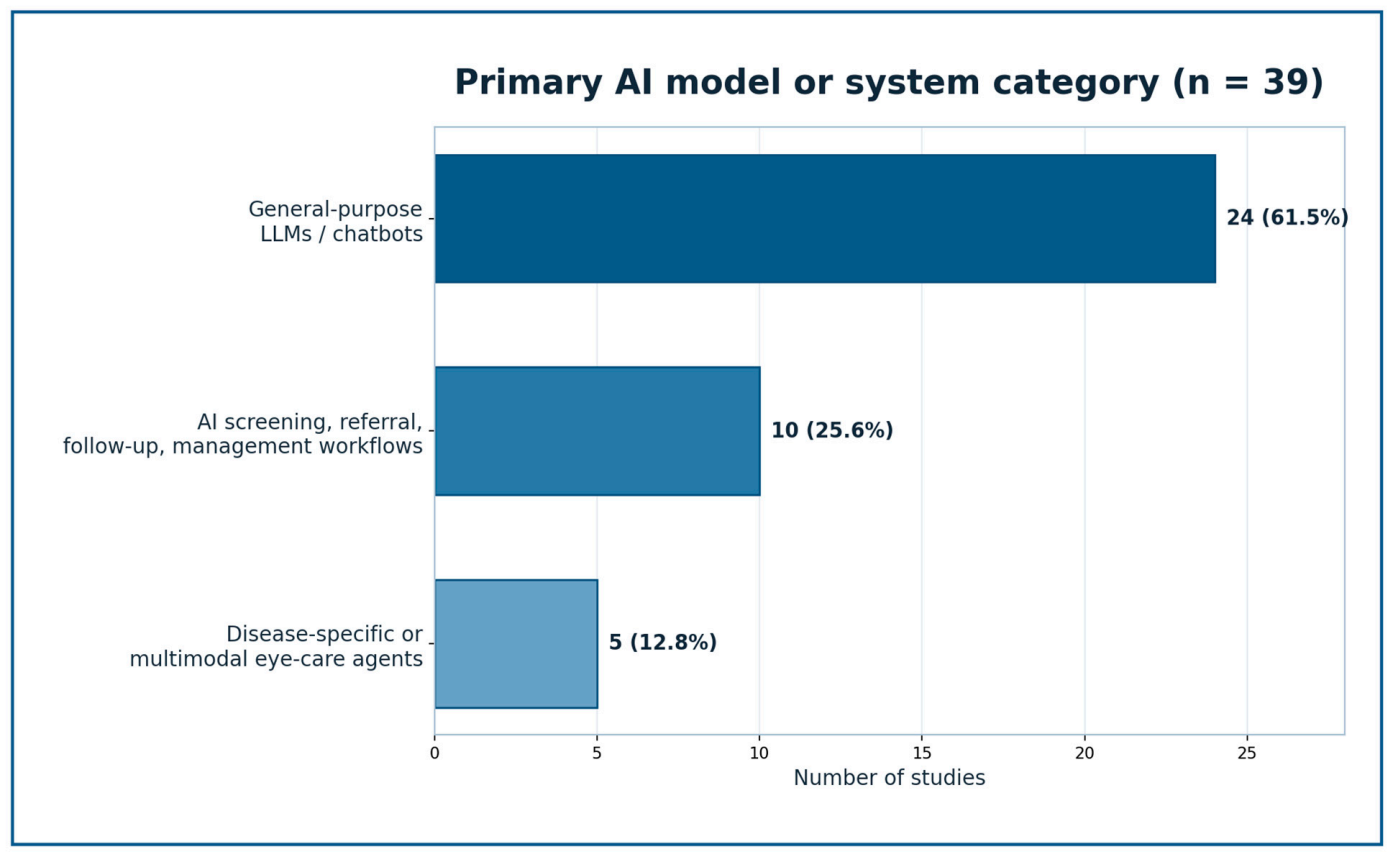
Primary AI model or system category represented in the final 39-study synthesis.

Large language models and chatbot-based systems were the most prominent primary AI category in the included evidence base, representing 24 of 39 studies (61.5%). AI screening, referral, follow-up, or management-support workflows accounted for 10 studies (25.6%), while disease-specific or multimodal eye-care agents accounted for five studies (12.8%). Frequently represented systems included ChatGPT-family models, Gemini/Bard, ChatGPT-4o, Microsoft Copilot/Bing, disease-specific AI agents, and ophthalmology-specific LLM or multimodal platforms. For Figure 3, each study was assigned to one primary AI model or system category based on the main AI application evaluated. The primary AI model or system categories are summarized in Figure 3.

**Figure 4:**
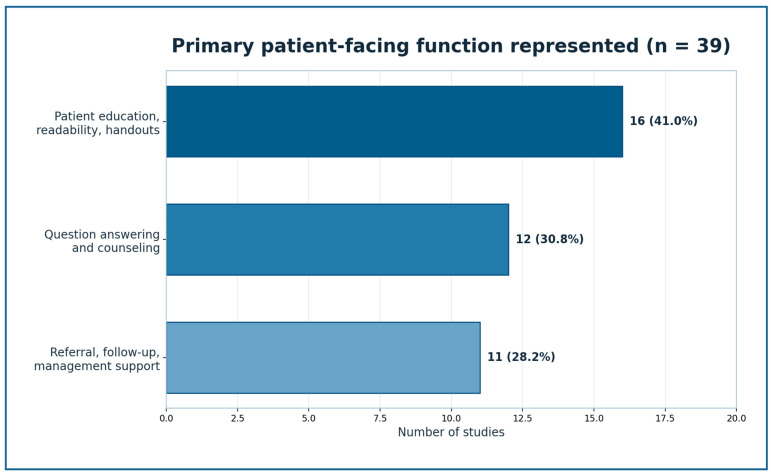
Primary patient-facing function represented in the final 39-study synthesis.

Patient-facing functions included answering common eye-care questions, generating patient educational materials, simplifying ophthalmic language, preparing plain-language summaries, supporting patient handouts, providing counseling-related responses, and supporting referral or follow-up workflows. The most common primary function was patient education, readability, or handout generation, reported in 16 of 39 studies (41.0%), followed by question answering and counseling in 12 studies (30.8%) and referral, follow-up, or management support in 11 studies (28.2%). For classification purposes, each study was assigned to one primary patient-facing function based on its main reported AI application; therefore, the counts in Figure 4 represent mutually exclusive study counts and sum to 39 studies. The retained management and screening studies were included only when their AI application was linked to referral, follow-up, or patient-facing management, not when the paper was limited to technical image classification alone. The primary patient-facing functions are summarized in Figure 4.

### 3.4. Outcome Reporting

**Figure 5:**
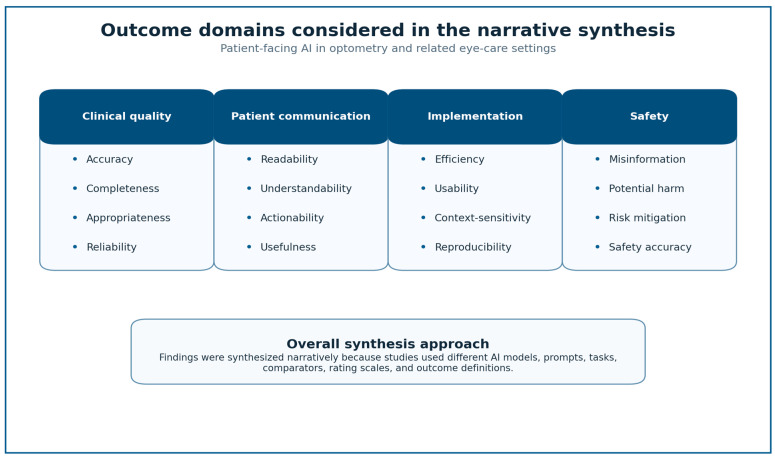
Outcome domains considered in the narrative synthesis of patient-facing AI studies.

Across the included 39 studies, the most emphasized outcome domains were accuracy, clinical quality, appropriateness, readability, completeness, understandability, and reliability of AI-generated information. Some studies also evaluated efficiency, actionability, usefulness, safety, potential harm, satisfaction, or trust. Because studies used different rubrics and did not apply a shared threshold for “acceptable” accuracy or readability, pooled acceptability estimates were not calculated. Instead, outcomes were interpreted as directional and domain-specific findings. Among the seven recent patient-facing LLM studies, six reported generally favorable or clinically acceptable accuracy, quality, or appropriateness with important qualifications, while all seven identified limitations related to readability, completeness, model dependence, prompt dependence, or the need for clinician review. Across the full 39-study evidence base, patient-centered outcomes such as satisfaction, trust, usability, adherence, behavioral change, and long-term clinical impact remained inconsistently reported. These outcomes were grouped narratively into clinical quality, patient communication, implementation, and safety domains, as summarized in Figure 5.

Figure 5 is intended as a narrative synthesis map rather than a pooled outcome model. Studies evaluating AI answers, patient handouts, or educational materials contributed mainly to the clinical-quality and patient-communication domains; studies evaluating referral, follow-up, triage, or screening-linked management contributed mainly to the implementation domain; and studies reporting misinformation, missing referral warnings, potentially harmful advice, or need for clinician oversight informed the safety domain. Because many publications assessed more than one domain, the domains should be interpreted as overlapping themes rather than mutually exclusive categories.

Study-quality appraisal using relevant JBI domains indicated heterogeneous methodological strength across the evidence base. Of the 39 included studies, seven (17.9%) were appraised as low concern, 29 (74.4%) as some concern, and three (7.7%) as high or unclear concern. These appraisal categories were not used to exclude studies, but they were used to calibrate interpretation: findings from low-concern randomized, real-patient, or implementation studies were interpreted as stronger applied evidence, whereas findings from simulated prompts, brief reports, correspondence-format evaluations, or studies with unclear sampling and comparator procedures were interpreted as preliminary or hypothesis-generating. This approach is summarized in Table 3 and provides the context for Figure 5.

Because the included studies differed substantially in design, task type, AI model, scoring rubric, and outcome scale, outcome patterns were synthesized narratively rather than pooled statistically. This approach avoided combining clinically heterogeneous accuracy, readability, usefulness, and safety outcomes into a single summary estimate.

### 3.5. Summary of Recent Patient-Facing LLM Studies

Several recent studies directly evaluated patient-facing LLM applications in ophthalmology and related eye-care settings. These studies are important because they move beyond general diagnostic AI and assess real patient questions, patient educational materials, readability, counseling quality, language adaptation, and clinical appropriateness. Table 4 provides a focused summary of seven recent patient-facing LLM studies published in 2025–2026, selected because they most directly represent contemporary counseling and education applications relevant to the review objective.

### 3.6. Narrative Synthesis of Reported Effectiveness and Limitations

Overall, the included studies suggest that AI-powered systems can generate useful, coherent, and often clinically relevant patient-facing information in eye-care contexts. However, performance was not uniform. The quality of AI-generated output varied by clinical topic, model version, prompt structure, language, reference standard, and evaluation criteria. Some studies reported favorable accuracy, appropriateness, or usefulness, whereas others identified incomplete, overly complex, potentially unsafe, or less readable outputs.

Across the included studies, measurable patient-level adverse events or observed unsafe behaviors were rarely reported. Reported drawbacks were therefore categorized narratively as potential misinformation, incomplete or overly complex responses, missing safety-netting or referral warnings, readability above patient education levels, and the need for clinician review before patient use.

Recent LLM studies support the potential role of AI as a drafting, simplification, or educational support tool. AI-generated patient handouts and answers to common patient questions may be useful, particularly when reviewed by clinicians. However, readability remained a recurring concern because some AI-generated responses were written above recommended patient education levels. In multilingual contexts, AI systems showed promise, but performance was not consistently superior to professional or society-developed patient education materials.

Chronic eye disease education, glaucoma counseling, diabetic retinopathy referral/follow-up, myopia education, cataract surgery counseling, and oculoplastic or retinal-condition patient questions were recurrent areas of evaluation. These topics are clinically important because patient understanding, adherence, symptom recognition, and follow-up behavior influence outcomes. The evidence supports AI as a supervised support tool, not as an autonomous source of clinical advice.

## 4. Discussion

This systematic review synthesized 39 eligible studies evaluating AI-powered systems for patient counseling, education, communication, referral/follow-up, and management-related support in optometry and related eye-care settings. The main message is not simply that AI can answer ophthalmic questions; rather, the evidence shows that AI performance depends strongly on the clinical task, model version, prompt, language, comparator, and outcome measure. Across the included studies, large language models and chatbot-based tools showed potential for producing patient-facing explanations and educational materials, but the evidence remains too heterogeneous to support unsupervised clinical use.

A focused interpretation is important because the objective of this review is different from broad AI-in-ophthalmology reviews. Diagnostic screening studies and technical deep-learning papers have shown that AI can perform well in image-based eye-care tasks, especially diabetic retinopathy detection [1,2]. However, patient counseling requires different evidence. A system used for counseling must provide correct information, communicate at an appropriate reading level, avoid unsafe reassurance, recognize when referral is needed, and support patient understanding without replacing professional judgment. Therefore, this review emphasizes patient-facing communication outcomes rather than technical diagnostic performance alone.

### 4.1. Principal Interpretation of the 39-Study Evidence Base

The retained evidence base suggests that AI may be useful as a supervised adjunct for common eye-care communication tasks. These tasks include answering frequently asked patient questions, preparing or simplifying educational materials, explaining chronic diseases such as glaucoma, diabetic retinopathy, myopia, and dry eye disease, supporting cataract and pediatric ophthalmology education, and reinforcing referral or follow-up instructions. This is clinically relevant because many eye-care conditions require repeated counseling over time, and patients often seek explanations outside the clinic through online resources.

The strength of the evidence is strongest for model-output evaluation and educational content generation, and weakest for real-world patient outcomes. Many studies assessed whether AI responses were accurate, complete, readable, or appropriate when judged by clinicians, experts, guidelines, or society patient information. Fewer studies examined whether patients actually understood the output, trusted it appropriately, followed advice safely, adhered to treatment, or attended follow-up. For this reason, the current review supports AI as an educational support tool, not as an independent counseling provider.

The findings also show that accuracy alone is not enough. An AI response may be factually acceptable but too long, too complex, insufficiently personalized, or missing a safety warning. Conversely, a readable response may omit important clinical nuance. Patient-facing quality therefore requires a combined assessment of accuracy, completeness, readability, actionability, safety, and clinical appropriateness. This combined standard is especially important in optometry and ophthalmology because symptoms such as flashes, floaters, painful red eye, sudden visual loss, postoperative symptoms, and worsening diabetic eye disease may require urgent referral rather than generic reassurance.

### 4.2. Comparison with Previous Literature and Reviews

The present review is consistent with the earlier ophthalmology AI literature in showing that AI has moved rapidly from diagnostic tasks toward broader clinical communication and education. Keskinbora and Guven and Costin et al. described the expanding role of AI in ophthalmology while emphasizing limitations related to transparency, reliability, ethics, and clinical implementation [3,4]. The present review extends that background by focusing on the patient-facing layer of care: whether AI-generated information can support counseling, education, question answering, follow-up communication, or management-related advice.

Several recent reviews of LLMs in ophthalmology and eye education also support the need for a cautious interpretation. Zhang et al. mapped LLM evaluations in ophthalmology and described substantial variability in models, tasks, subspecialties, and outcome measures [45]. Agnihotri et al. similarly highlighted rapid growth in ophthalmology LLM publications but noted that evaluation methods remain inconsistent [46]. Artiaga et al. described potential utility for clinicians, patients, researchers, and educators, while Li et al., Andrew and Rodenko, and See et al. emphasized the need for validation, safety assessment, and clearer reporting before routine use in eye-care education [47,48,49,50]. The current review adds a narrower contribution by separating patient-facing counseling and education from broad diagnostic or technical AI applications.

The patient-facing primary studies included in this review are also broadly consistent with the review literature. Studies of common ophthalmology questions, glaucoma education, pediatric ophthalmology materials, AI-generated handouts, and ophthalmologist-versus-chatbot responses suggest that AI outputs can be useful, but not uniformly reliable or patient-ready [38,39,40,41,42,43,44]. Some studies found favorable accuracy or completeness, whereas others reported concerns about readability, length, quality variation, missing warnings, or inferior performance compared with society-developed educational materials. This pattern supports a balanced conclusion: AI can assist clinicians in preparing or reviewing educational content, but outputs should be checked before they are given to patients. Table 5 summarizes how the present review compares with the key previous literature and selected patient-facing studies.

### 4.3. Accuracy, Completeness, and Clinical Appropriateness

Accuracy was the most visible outcome across the included studies, but it was measured in different ways. Some studies used expert ratings, some compared answers with professional guidelines or society materials, and others used study-specific rubrics. This makes direct pooling inappropriate, but the pattern is clinically useful: AI systems can produce correct and coherent responses in many eye-care topics, yet errors, omissions, overgeneralized advice, and variation between models remain important concerns.

This finding agrees with broader LLM reviews in ophthalmology, which describe encouraging performance but emphasize that evaluation frameworks are not standardized [45,46,47,48,49,50]. For patient counseling, variability is especially important because incomplete or overconfident answers may affect when patients seek care. An AI answer about cataract surgery, glaucoma drops, diabetic retinopathy follow-up, pediatric surgery, or retinal detachment symptoms may appear clear while still missing individualized risk factors or urgent referral criteria. Therefore, clinical appropriateness should be judged not only by factual correctness but also by whether the response is safe for patient behavior.

The review also suggests that model comparisons should be interpreted carefully. No model family consistently outperformed all others across ophthalmic tasks. ChatGPT-family models, including newer GPT-4o-based evaluations, often produced clinically useful patient-facing responses, but studies also reported concerns about length, readability, incompleteness, or missing safety warnings. Gemini/Bard responses were generally comparable in some tasks but sometimes scored lower than ChatGPT-family systems depending on language, prompt, and scoring rubric. Disease-specific and multimodal systems may offer advantages when they are designed for a narrow task, such as myopia consultation, fundus-fluorescein angiography question answering, or ophthalmology-specific patient inquiries, but these advantages should not be generalized beyond the validated task and dataset. Therefore, model choice for clinical education should be guided by the clinical topic, language, patient population, prompting method, comparator, and requirement for clinician review rather than by model name alone.

Readability and patient-education quality should be interpreted alongside accuracy. Several included studies showed that AI can simplify ophthalmic terminology and generate structured educational materials, but responses may still be too long, too technical, or above recommended patient education levels. Therefore, AI-generated counseling should be judged not only by factual correctness but also by completeness, understandability, actionability, and safety for patient behavior.

Multilingual and culturally appropriate communication also require attention. Studies in Turkish and Traditional Chinese settings show that AI evaluation cannot be limited to English-language prompts [39,42]. Eye-care counseling often occurs in multilingual contexts, and literal translation may not preserve clinical nuance, urgency, or health-literacy appropriateness. Future research should test AI-generated materials in the languages and cultural contexts in which patients will actually use them.

### 4.4. Patient-Centered Outcomes and Real-World Implementation

A major weakness of the current evidence is the limited measurement of patient-centered outcomes. Many studies evaluated AI outputs rather than patient outcomes. As a result, the literature can tell us whether expert reviewers considered an answer accurate or readable, but it often cannot tell us whether patients understood the answer, trusted it appropriately, followed instructions, attended follow-up, avoided unnecessary anxiety, or avoided delayed care.

This limitation is not unique to the present review; recent ophthalmology LLM reviews also emphasize that real-world evaluation remains underdeveloped [45,46,47,48,49,50]. For optometry and eye-care practice, this gap is clinically significant. Counseling success is not measured only by the content of the message; it is measured by whether the patient can use the information safely. A future evidence base should therefore include prospective patient studies, pragmatic clinical evaluations, and outcomes such as understanding, recall, satisfaction, trust calibration, adherence, follow-up attendance, escalation of urgent symptoms, and clinician workload.

The role of AI in practice should therefore be framed as supportive rather than substitutive. Evidence-based conclusions from the included studies support the potential of AI to draft, simplify, or structure patient-facing educational content under supervision. By contrast, recommendations for governance, workflow integration, autonomous deployment, adherence support, and long-term follow-up remain conceptual or implementation-oriented because prospective patient studies and real-world clinical deployment data remain limited. AI may help clinicians draft educational explanations, generate first versions of patient handouts, simplify jargon, prepare multilingual materials, or provide structured answers that clinicians can review, but the present evidence does not support unsupervised use as a substitute for clinician-led counseling.

### 4.5. Safety, Governance, and Clinical Accountability

Safety was inconsistently reported across the included studies, even though it is one of the most important issues for patient-facing AI. Potential safety concerns include misinformation, incomplete warnings, inappropriate reassurance, failure to recommend urgent referral, overconfident statements, hallucinated details, and advice that is not individualized to patient history. In eye care, these concerns are clinically important because delayed assessment may lead to avoidable vision loss in conditions such as retinal detachment, acute glaucoma, severe ocular infection, trauma, or proliferative diabetic retinopathy.

The discussion in broader AI reviews is therefore directly relevant to this manuscript. Previous reviews emphasize that clinical AI requires governance, transparency, accountability, monitoring, and human oversight [3,4,45,46,47,48,49,50]. For patient education, this means that AI output should be reviewed before being integrated into clinical workflows, especially when advice involves medications, surgery, urgent symptoms, pediatric care, or chronic disease follow-up. Clinics should also clarify to patients that AI-generated information is educational and does not replace personalized assessment by a qualified eye-care professional.

Future implementation should include safeguards such as approved prompt templates, clinician review, clear referral warnings, reading-level targets, version tracking, documentation of model updates, reporting of unsafe outputs, and periodic revalidation. These safeguards are necessary because AI models change over time, and findings from one version may not remain valid after updates or changes in deployment conditions.

### 4.6. Strengths and Limitations

This review has several strengths. It focuses on a clinically important and rapidly developing area: AI-supported patient counseling, education, and management-related communication in optometry and related eye-care settings. It separates patient-facing communication studies from purely technical image-classification or segmentation studies, which makes the conclusions more relevant to counseling practice. It also synthesizes multiple outcome domains, including accuracy, readability, completeness, appropriateness, safety, usefulness, and patient-centered outcomes when available.

The review also has limitations. First, the included studies were highly heterogeneous in AI model, prompt design, disease area, language, comparator, scoring rubric, and outcome definition. This limited direct comparison between studies and prevented quantitative pooling. Second, many studies were based on simulated prompts, AI-generated outputs, or expert review rather than real patient encounters. Third, reporting of participant characteristics, setting, model version, prompt wording, and evaluator background was incomplete in several studies. Fourth, patient-centered outcomes such as satisfaction, trust, usability, adherence, follow-up behavior, and clinical impact were not consistently measured. Finally, because AI tools evolve rapidly, results may become outdated when models are updated. Although methodological quality was appraised using JBI tools, the diversity of study designs and outcome measures meant that appraisal findings were interpreted narratively rather than used to exclude studies or produce a pooled quality score.

### 4.7. Implications for Future Research

Future studies should move from output-only evaluations toward prospective and real-world designs. Studies should test AI tools with real patients, caregivers, and clinicians in realistic counseling scenarios. They should use standardized rubrics for accuracy, completeness, appropriateness, readability, actionability, safety, and potential harm. They should also report model version, query date, prompt wording, language, clinical context, reference standard, evaluator expertise, and whether responses were regenerated or edited. Future evaluations should also assess accessibility for low-vision users, including screen-reader compatibility, readable formatting, adequate visual contrast, plain-language structure, and the suitability of AI-generated patient education materials for patients with visual impairment.

Patient-centered outcomes should become a priority. Future research should measure whether AI improves patient understanding, reduces confusion, supports adherence, improves follow-up attendance, increases appropriate care-seeking, or reduces clinician workload without compromising safety. Multilingual studies and low-health-literacy evaluations are especially important for equitable implementation. In the meantime, AI in optometry and related eye-care settings should be used as a clinician-supervised support tool for education and communication rather than an autonomous replacement for professional counseling.

## 5. Conclusions

This systematic review included 39 studies focused on patient-facing AI applications for counseling, education, communication, referral/follow-up, or management support in optometry and related eye-care settings. AI-powered systems, particularly LLMs and chatbot-based tools, show meaningful potential as supervised adjuncts for patient education and management-related communication. However, the current evidence is derived primarily from expert evaluations, simulated prompts, AI-generated outputs, content comparisons, and limited clinical pathway studies rather than from prospective real-world deployment with patient-centered outcomes. Therefore, conclusions about accuracy, readability, clinical appropriateness, and usefulness should be interpreted cautiously, and recommendations for implementation should be viewed as future-facing until validated in real patients, diverse languages, and routine clinical settings. Before routine adoption, studies should use standardized evaluation frameworks, report model and prompt details transparently, monitor safety and potential harm, and measure patient understanding, trust, adherence, follow-up behavior, and clinical outcomes.

## Figures and Tables

**Table 1:** Eligibility framework.

Domain	Eligibility Definition
Population	Ophthalmic or optometric patients, caregivers, simulated patient profiles, patient questions, patient-facing educational materials, or eye-care clinical contexts.
Intervention	AI-powered systems, including LLMs, chatbots, machine-learning tools, deep-learning models, rule-based systems, or hybrid systems used for counseling, education, communication, referral, follow-up, or management support.
Comparator	Human counseling, clinician or expert assessment, professional guidelines, society patient education materials, reference standards, or other AI systems.
Outcomes	Accuracy, readability, completeness, appropriateness, reliability, usefulness, satisfaction, trust, usability, safety, potential harm, actionability, and other patient-centered or communication-related outcomes.
Exclusions	Reviews, commentaries, letters, response articles, non-eye-care AI studies, examination-only studies, and purely technical classification/segmentation/prediction studies without patient-facing relevance.

**Table 2:** Main evidence domains represented in the 39 included studies.

Domain	Examples in the Retained Evidence Base
Patient education and readability	Dry eye disease, cataract surgery, myopia, glaucoma, pediatric ophthalmology, AAO/AAPOS materials, medical-jargon translation, patient handouts.
Question answering and counseling	Cataract counseling, retinal detachment information, oculoplastic patient queries, choroidal melanoma support, real ophthalmology patient questions, glaucoma counseling, herpes zoster ophthalmicus education.
Referral, follow-up, and management support	At-risk referral from primary care to eye specialists, diabetic retinopathy screening/follow-up, AI-human hybrid teleophthalmology workflows, strabismus screening/management, geographic atrophy prediction/management.
Multilingual and accessibility-focused studies	Traditional Chinese glaucoma counseling, Turkish glaucoma education, Spanish-language patient handouts, simplification of clinical notes and medical terminology.
Model families	ChatGPT family models, Gemini/Bard, ChatGPT-4o, Microsoft Copilot/Bing, EyeGPT, ChatMyopia, FFA-GPT, and other ML or hybrid systems used in patient-facing pathways.

**Table 3:** Summary of methodological appraisal, evidence grouping, and interpretation approach in the final 39-study synthesis.

Evidence or Appraisal Dimension	Summary in the 39-Study Synthesis	Influence on Interpretation
Overall JBI appraisal	Low concern: 7/39 (17.9%); some concern: 29/39 (74.4%); high/unclear concern: 3/39 (7.7%).	Low-concern studies were treated as stronger support; some-concern and high/unclear studies were interpreted cautiously.
Primary model/system category	General-purpose LLMs/chatbots: 24/39 (61.5%); AI screening/referral/follow-up/management workflows: 10/39 (25.6%); disease-specific or multimodal agents: 5/39 (12.8%).	Model comparisons were interpreted by task and context rather than as evidence that one model family is universally superior.
Primary patient-facing function	Patient education, readability, or handout generation: 16/39 (41.0%); question answering and counseling: 12/39 (30.8%); referral, follow-up, or management support: 11/39 (28.2%).	Evidence was synthesized by function because counseling, education, and follow-up tools had different comparators and outcomes.
Outcome interpretation	Accuracy, clinical quality, appropriateness, readability, completeness, and reliability were common output-level outcomes; patient-centered and behavioral outcomes were reported less consistently.	Output-only or expert-evaluated findings were not treated as proof of real-world clinical effectiveness.
Implementation inference	Prospective patient deployment and long-term clinical outcomes were limited across the literature.	Recommendations for governance, safety monitoring, and real-patient validation were framed as future implementation requirements rather than proven effects.

**Table 4:** Selected recent patient-facing AI studies included in the review.

Study	AI/Focus	Main Finding	DOI
Riazi Esfahani et al., 2025 [38]	Common ophthalmology questions	ChatGPT responses were generally favorable for accuracy/comprehensiveness/clarity, but readability remained above the recommended level for patient education.	10.7759/cureus.87920
Dal et al., 2026 [39]	Turkish glaucoma patient education	LLMs produced useful educational content, but readability and comprehensiveness differed by model and prompting approach.	10.14744/eer.2025.93723
Yang et al., 2025 [40]	Pediatric ophthalmologic surgery materials	AI-generated pediatric surgical education materials were not consistently superior to AAPOS materials on quality domains.	10.3928/01913913-20250404-01
Bondok et al., 2025 [41]	AI chatbot versus ophthalmologist responses	ChatGPT-4o responses were comparable in accuracy and empathy, but tended to be longer and less readable than clinician responses.	10.2147/OPTH.S549820
Lin et al., 2026 [42]	Glaucoma counseling in Traditional Chinese	ChatGPT and Gemini gave reasonably appropriate responses, with ChatGPT scoring higher overall than Gemini.	10.4103/tjo.TJO-D-25-00058
Wu et al., 2026 [43]	ChatGPT-4o patient handouts versus AAO materials	ChatGPT-4o generated handouts competitive with AAO materials, including notable findings in Spanish-language materials.	10.1167/tvst.15.2.14
Hamzeh et al., 2026 [44]	Real ophthalmology patient questions	Most ChatGPT-4o responses were accurate and complete, but some were incomplete or unacceptable, and readability remained high.	10.1016/j.xops.2025.101007

**Table 5:** Comparison of the present review with the key previous literature and patient-facing studies.

Reference	Main Contribution	Comparison with This Review
Gulshan et al. [1]	AI performed strongly for diabetic retinopathy image screening.	Useful background only; counseling safety needs separate evidence.
Abramoff et al. [2]	Autonomous diabetic retinopathy detection in a clinical pathway.	Supports feasibility of AI-supported referral/screening, not unsupervised counseling.
Keskinbora and Guven [3]	Broad review of AI in ophthalmology.	Supports rationale for studying patient-facing AI in eye care.
Costin et al. [4]	Reviewed advantages and limitations of ophthalmic AI.	Aligns with cautious conclusion and need for validation.
Chalasani et al. [5]	Discussed structured evaluation of AI in healthcare practice.	Supports standardized assessment of counseling tools.
Zhang et al. [45]	Systematic review of LLMs in ophthalmology.	Confirms rapid growth and heterogeneity; this review is narrower and patient-facing.
Agnihotri et al. [46]	Review of LLM publications in ophthalmology journals.	Supports need for clear model, prompt, and outcome reporting.
Artiaga et al. [47]	Scoping review of LLM utility for clinicians, patients, researchers, and educators.	This review adds focus on counseling and education outcomes.
Li et al. [48]	Systematic review of LLMs in primary care ophthalmic education.	Closely aligned with education focus; this review also includes referral/follow-up support.
Andrew and Rodenko [49]	Narrative review of LLMs and eye education.	Supports emphasis on readability, health literacy, and safety.
See et al. [50]	Scoping review of ophthalmology LLM use-cases.	Supports governance and implementation safeguards.
Riazi Esfahani et al. [38]	Assessed ChatGPT accuracy and readability for common ophthalmology questions.	Direct evidence that accuracy and readability must be assessed together.
Dal et al. [39]	Assessed Turkish glaucoma patient education materials.	Shows need for multilingual chronic-disease counseling validation.
Yang et al. [40]	Assessed pediatric ophthalmology surgery education materials.	Shows AI material is not always superior to society material.
Bondok et al. [41]	Compared ophthalmologist and chatbot responses to patient questions.	Supports supervised drafting, not autonomous replacement.
Lin et al. [42]	Evaluated glaucoma counseling in Traditional Chinese.	Shows language-specific validation is important.
Wu et al. [43]	Compared ChatGPT-4o handouts with AAO materials.	Supports use as content-generation aid with expert review.
Hamzeh et al. [44]	Evaluated ChatGPT-4o accuracy and readability for patient questions.	Reinforces need for both clinical and readability review.

## Data Availability

The data supporting this systematic review are included in the article and Supplementary Materials. Further inquiries can be directed to the corresponding authors.

## Supplementary Materials

The following supporting information can be downloaded at: https://www.mdpi.com/article/10.3390/healthcare14152270/s1, File S1. PRISMA 2020 Checklist and Search Strategy. Table S1. Final Included Studies and DOI/Stable Identifier Audit (*n* = 39). Table S2. Study-by-study JBI methodological quality appraisal of the 39 included studies. All included studies listed in the Supplementary Materials are represented in the main reference list.
