# Supplementary material for: Accuracy and Effectiveness of AI-Powered Systems in Patient Counseling, Education, and Management in Optometry and Related Eye-Care Settings: A Systematic Review
Source: Healthcare (Basel). 2026 Jul 24;14(15):2270. doi: 10.3390/healthcare14152270 (PMC13464863; doi:10.3390/healthcare14152270)
Supplement: Supplementary file 1 [file healthcare-14-02270-s001.zip › Supplementary File S1 PRISMA Checklist and Search Strategy FINAL 24 JULY.pdf]

Supplementary File S1. PRISMA 2020 Checklist and Search Strategy

Manuscript title: Accuracy and Effectiveness of AI-Powered Systems in Patient Counseling, Education, and Management in Optometry and Related Eye-Care Settings: A Systematic Review

PROSPERO registration: CRD420251234880

Section A. PRISMA 2020 Checklist

PRISMA item	Topic	Checklist detail	Location in manuscript/supplement
**1**	Title	Identified the report as a systematic review.	Title page
**2**	Abstract	Structured summary including background/objectives, methods, results, and conclusions.	Abstract
**3**	Rationale	Rationale for reviewing patient-facing AI in optometry and related eye-care settings.	Introduction
**4**	Objectives	Review objective and outcomes stated.	Introduction, Clinical Significance, and review question
**5**	Eligibility criteria	Eligible populations, interventions, comparators, outcomes, designs, and exclusions described.	Methods 2.3 and Table 1
**6**	Information sources	Databases and time limits reported.	Methods 2.2; Supplementary File S1, Section B
**7**	Search strategy	Database search concepts and adapted search strings provided.	Supplementary File S1, Section B
**8**	Selection process	Two-reviewer selection process and disagreement resolution described.	Methods 2.4
**9**	Data collection process	Structured extraction and two-reviewer process described.	Methods 2.4
**10**	Data items	Study, AI model, comparator, function, outcome, values, and traceability fields described.	Methods 2.4
**11**	Study risk of bias / quality assessment	JBI critical appraisal domains used according to study design.	Methods 2.4; Results 3.4; Table 3; Supplementary Table S2
**12**	Effect measures	Not applicable for pooled effect estimates; outcomes were extracted as reported.	Methods 2.6
**13**	Synthesis methods	Narrative/descriptive synthesis and no quantitative pooling due to heterogeneity.	Methods 2.6; Results 3.2-4.6

**14**	Reporting bias assessment	Formal reporting-bias assessment not performed; reporting limitations considered narratively.	Results 3.4; Discussion 4.6-4.7; Supplementary Table S2
**15**	Certainty assessment	Certainty assessment was not undertaken.	Methods 2.6; Discussion 4.6
**16a**	Study selection results	Records identified, excluded, assessed, and included reported.	Results 3.1 and Figure 1
**16b**	Excluded studies	Full-text exclusion reasons summarized in PRISMA flow diagram.	Results 3.1 and Figure 1
**17**	Study characteristics	Characteristics and evidence domains described.	Results 3.2; Tables 2 and 4; Supplementary Table S1
**18**	Quality appraisal results	JBI appraisal findings summarized narratively.	Results 3.4; Table 3; Supplementary Table S2
**19**	Results of individual studies	Selected studies and findings summarized.	Table 4; Table 5; Supplementary Table S1
**20**	Synthesis results	Narrative synthesis of model categories, functions, outcomes, and effectiveness.	Results 3.2-3.6; Figures 2-5; Tables 3-4
**21**	Reporting biases	Potential reporting and outcome-reporting limitations discussed.	Discussion 4.6-4.7
**22**	Certainty of evidence	Not formally assessed; implications discussed cautiously.	Discussion 4.6-4.7
**23**	Discussion	Interpretation, comparison with prior literature, limitations, and implications.	Discussion
**24a**	Registration	PROSPERO registration number reported.	Methods 2.1
**24b**	Protocol availability	Protocol/search information supplied in supplementary materials.	Methods 2.1; Supplementary File S1
**24c**	Protocol amendments	Protocol amendments were recorded in PROSPERO. The main amendments clarified the review title and scope, search date range, quality appraisal approach, and synthesis plan. Meta-analysis was not performed because of heterogeneity, and findings were synthesized narratively.	Methods 2.1 and 2.6; PROSPERO record
**25**	Support	Funding and funder role described.	Funding; Acknowledgments
**26**	Competing interests	Conflict-of-interest statement included.	Conflicts of Interest

**27**	Availability of data, code, and materials	Supplementary materials and data availability statement included.	Data Availability; Supplementary Materials

Section B. Database Search Strategy

Search period: 1 January 2020 to 31 May 2026. Databases: PubMed, Embase, Web of Science, Cochrane Library, and IEEE Xplore. Only published studies were sought; no language restriction was applied. Search strings were adapted to each database interface and combined terms for artificial intelligence, large language models, chatbots, machine learning, optometry, ophthalmology, eye care, patient counseling, patient education, communication, referral, follow-up, and management support.

Database	Search strategy / adaptation
**PubMed**	(("Artificial Intelligence"[Mesh] OR "Machine Learning"[Mesh] OR "Deep Learning"[Mesh] OR artificial intelligence[tiab] OR machine learning[tiab] OR deep learning[tiab] OR large language model*[tiab] OR LLM*[tiab] OR ChatGPT[tiab] OR chatbot*[tiab] OR generative AI[tiab] OR Gemini[tiab] OR Bard[tiab] OR Copilot[tiab]) AND (optometr*[tiab] OR ophthalmolog*[tiab] OR eye care[tiab] OR ocular[tiab] OR retina*[tiab] OR glaucoma[tiab] OR cataract*[tiab] OR diabetic retinopathy[tiab] OR myopia[tiab] OR dry eye[tiab] OR strabismus[tiab]) AND (patient education[tiab] OR patient counseling[tiab] OR counselling[tiab] OR communication[tiab] OR question answer*[tiab] OR readability[tiab] OR understandability[tiab] OR handout*[tiab] OR referral[tiab] OR follow-up[tiab] OR management[tiab])) Filters: 2020/01/01-2026/05/31.
**Embase**	('artificial intelligence'/exp OR 'machine learning'/exp OR 'deep learning'/exp OR 'large language model':ti,ab OR llm*:ti,ab OR chatgpt:ti,ab OR chatbot*:ti,ab OR gemini:ti,ab OR bard:ti,ab OR copilot:ti,ab) AND ('ophthalmology'/exp OR 'optometry'/exp OR 'eye disease'/exp OR optometr*:ti,ab OR ophthalmolog*:ti,ab OR 'eye care':ti,ab OR ocular:ti,ab OR glaucoma:ti,ab OR cataract*:ti,ab OR 'diabetic retinopathy':ti,ab OR myopia:ti,ab OR strabismus:ti,ab) AND ('patient education'/exp OR 'counseling'/exp OR communication:ti,ab OR 'question answering':ti,ab OR readability:ti,ab OR handout*:ti,ab OR referral:ti,ab OR 'follow

	up':ti,ab OR management:ti,ab) AND [2020-2026]/py.
**Web of Science**	TS=(("artificial intelligence" OR "machine learning" OR "deep learning" OR "large language model*" OR LLM* OR ChatGPT OR chatbot* OR Gemini OR Bard OR Copilot) AND (optometr* OR ophthalmolog* OR "eye care" OR ocular OR retina* OR glaucoma OR cataract* OR "diabetic retinopathy" OR myopia OR "dry eye" OR strabismus) AND ("patient education" OR counsel* OR communication OR "question answering" OR readability OR understandability OR handout* OR referral OR follow-up OR management)). Timespan: 2020-2026.
**Cochrane Library**	([artificial intelligence] OR [machine learning] OR [deep learning] OR [large language model] OR [ChatGPT] OR [chatbot]) AND ([optometry] OR [ophthalmology] OR [eye care] OR [glaucoma] OR [cataract] OR [diabetic retinopathy] OR [myopia] OR [strabismus]) AND ([patient education] OR [counseling] OR [communication] OR [question answering] OR [readability] OR [referral] OR [follow-up] OR [management]). Date range: 2020 to 31 May 2026.
**IEEE Xplore**	("artificial intelligence" OR "machine learning" OR "deep learning" OR "large language model" OR ChatGPT OR chatbot) AND (optometry OR ophthalmology OR "eye care" OR ocular OR "diabetic retinopathy" OR glaucoma OR cataract OR myopia OR strabismus) AND (patient OR counseling OR education OR communication OR referral OR follow-up OR management). Date range: 2020-2026.
