# Supplementary material for: Accuracy and Effectiveness of AI-Powered Systems in Patient Counseling, Education, and Management in Optometry and Related Eye-Care Settings: A Systematic Review
Source: Healthcare (Basel). 2026 Jul 24;14(15):2270. doi: 10.3390/healthcare14152270 (PMC13464863; doi:10.3390/healthcare14152270)
Supplement: Supplementary file 1 [file healthcare-14-02270-s001.zip › Supplementary Table S1 FINAL 24 JULY.pdf]

Supplementary Table S1. Final Included Studies and DOI/Stable Identifier Audit (n = 39)

Manuscript title: Accuracy and Effectiveness of AI-Powered Systems in Patient Counseling, Education, and Management in Optometry and Related Eye-Care Settings: A Systematic Review

Study no.	Manuscript reference number	Included study reference	DOI / stable identifier
1	[6]	Young JA, Chang CW, Scales CW, Menon SV, Holy CE, Blackie CA. Machine learning methods using artificial intelligence deployed on electronic health record data for identification and referral of at-risk patients from primary care physicians to eye care specialists: retrospective, case-controlled study. JMIR AI. 2024;3:e48295.	10.2196/48295
2	[7]	Dihan QA, Brown AD, Chauhan MZ, Alzein AF, Abdelnaem SE, Kelso SD, et al. Leveraging large language models to improve patient education on dry eye disease. Eye (Lond). 2025;39(6):1115-1122.	10.1038/s41433-024-03476-5
3	[8]	Wei L, Mohammed ISK, Francomacaro S, Munir WM. Evaluating text-based generative artificial intelligence models for patient information regarding cataract surgery. Journal of Cataract and Refractive Surgery. 2024;50(1):95-96.	10.1097/j.jcrs.0000000000001288
4	[9]	Wu Y, Chen X, Zhang W, Liu S, Sum WMR, Wu X, et al. ChatMyopia: an AI agent for myopia-related consultation in primary eye care settings. iScience. 2025;28(11):113768.	10.1016/j.isci.2025.113768
5	[10]	Yilmaz İE, Berhuni M, Özer Özcan Z, Doğan L. Chatbots talk strabismus: can AI become the new patient educator? International Journal of Medical Informatics. 2024;191:105592.	10.1016/j.ijmedinf.2024.105592
6	[11]	Wu H, Su Z, Pan X, Shao A, Xu Y, Wang Y, et al. Enhancing diabetic retinopathy query responses: assessing large language model in ophthalmology. British Journal of Ophthalmology. 2025;109(11):1272-1278.	10.1136/bjo-2024-325861
7	[12]	Wu D, Li Y, Zhang H, Yang X, Mao Y, Chen B, et al. An artificial intelligence platform for the screening and managing of strabismus. Eye (Lond). 2024;38(16):3101-3107.	10.1038/s41433-024-03228-5
8	[13]	Armadá-Maresca F, Capote-Díaz P, Caminos-Melguizo A, Baoud-Ould-Haddi I. Clinical performance of an interactive platform based on artificial intelligence in ophthalmology: experience in a third-level reference center. Frontiers in Medicine. 2025;12:1593556.	10.3389/fmed.2025.1593556
9	[14]	Strzalkowski P, Strzalkowska A, Chhablani J, Pfau K, et al. Evaluation of the accuracy and readability of ChatGPT-4 and Google Gemini in providing information on retinal detachment: a multicenter expert comparative study. International Journal of Retina and Vitreous. 2024;10:61.	10.1186/s40942-024-00579-9
10	[15]	Tailor PD, D'Souza HS, Castillejo Becerra CM, Dahl HM, Patel NR, Kaplan TM, et al. Evaluation of AI summaries on interdisciplinary understanding of ophthalmology notes. JAMA Ophthalmology. 2025;143(5):410-419.	10.1001/jamaophthalmol.2025.0351
11	[16]	Raghu K, Tamilselvi S, Devishamani CS, Suchetha M, Rajalakshmi R, Raman R. The utility of ChatGPT in diabetic retinopathy risk assessment: a comparative study with clinical diagnosis. Clinical Ophthalmology. 2023;17:4021-4031.	10.2147/OPTH.S435052
12	[17]	Wolf RM, Channa R, Liu TYA, Zehra A, Bromberger L, Patel D, et al. Autonomous artificial intelligence increases screening and follow-up for diabetic retinopathy in youth: the ACCESS randomized control trial. Nature Communications. 2024;15:421.	10.1038/s41467-023-44676-z
13	[18]	Dow ER, Khan NC, Chen KM, Mishra K, Perera C, Narala R, et al. AI-human hybrid workflow enhances teleophthalmology for the detection of diabetic retinopathy. Ophthalmology Science. 2023;3(4):100330.	10.1016/j.xops.2023.100330
14	[19]	Shruthee SG, Vellingiri D, Rajagopal K, Shanmugaiah P. Diagnostic accuracy of an artificial intelligence based mHealth intervention for cataract detection: a multi-center prospective study in Tamil Nadu, India. National Journal of Community Medicine. 2025;16(09):887-894.	10.55489/njcm.160920255469
15	[20]	Gupta AS, Sulewski ME, Armenti ST. Performance of ChatGPT in cataract surgery counseling. Journal of Cataract and Refractive Surgery. 2024;50(4):424-425.	10.1097/j.jcrs.0000000000001345
16	[21]	Yigen İritaş S, İritaş İ. AI as a patient educator: evaluating ChatGPT's role in disseminating information on herpes zoster ophthalmicus. The European Research Journal. 2025;11(4):769-775.	10.18621/eurj.1616435
17	[22]	Ma R, Cheng Q, Yao J, Peng Z, Yan M, Lu J, et al. Multimodal machine learning enables AI chatbot to diagnose ophthalmic diseases and provide high-quality medical responses. npj Digital Medicine. 2025;8:64.	10.1038/s41746-025-01461-0
18	[23]	Mathenge W, Whitestone N, Nkurikiye J, Patnaik JL, Piyasena P, Uwaliraye P, et al. Impact of artificial intelligence assessment of diabetic retinopathy on referral service uptake in a low-resource setting: the RAIDERS randomized trial. Ophthalmology Science. 2022;2(4):100168.	10.1016/j.xops.2022.100168

Study no.	Manuscript reference number	Included study reference	DOI / stable identifier
19	[24]	Mereddy S, Polaki S, Aswini Kumar M, Mohanababu Ch, Prathibha Malini K, Sujatha D. AI and IoT-integrated smart devices for enhanced vision screening and diagnosis in eye care. In: 2024 2nd International Conference on Signal Processing, Communication, Power and Embedded Systems (SCOPES). IEEE; 2024.	10.1109/SCOPES64467.2024.10991151
20	[25]	Reiter GS, Lachinov D, Bühl W, Weigert G, Grechenig C, Mai J, et al. A novel management challenge in age-related macular degeneration: artificial intelligence and expert prediction of geographic atrophy. Ophthalmology Retina. 2025;9(5):421-430.	10.1016/j.oret.2024.10.029
21	[26]	Karataş E, Durmaz Engin C. From accuracy to comprehensibility: evaluating large language models for myopia patient queries. Health Policy and Technology. 2025;14(6):101073.	10.1016/j.hlpt.2025.101073
22	[27]	Dow ER, Chen KM, Zhao CS, et al. Artificial intelligence improves patient follow-up in a diabetic retinopathy screening program. Clinical Ophthalmology. 2023;17:3323-3330.	10.2147/OPTH.S422513
23	[28]	Al-Sharif EM, Penteado RC, Atwan M, et al. Evaluating the accuracy of ChatGPT and Google BARD in fielding oculoplastic patient queries: a comparative study on artificial versus human intelligence. Ophthalmic Plastic and Reconstructive Surgery. 2024;40(3):e95-e100.	10.1097/IOP.0000000000002567
24	[29]	Kufta AY, Djalilian A, et al. Enhancing patient education with AI: a readability analysis of AI-generated versus American Academy of Ophthalmology online patient education materials. Journal of Clinical Medicine. 2025;14(19):6968.	10.3390/jcm14196968
25	[30]	Balas M, Kaplan AJ, Esmail K, et al. Translating ophthalmic medical jargon with artificial intelligence: a comparative comprehension study. Canadian Journal of Ophthalmology. 2025;60(4):e602-e609.	10.1016/j.jcjo.2024.11.003
26	[31]	Cao Y, Lu W, Shi R, Liu F, Liu S, Xu X, et al. Performance of popular large language models in glaucoma patient education: a randomized controlled study. Advances in Ophthalmology Practice and Research. 2025;5(2):88-94.	10.1016/j.aopr.2024.12.002
27	[32]	Li G, Lin J, Akpek EK, et al. Enhancing online cataract surgery patient education materials through artificial intelligence. Canadian Journal of Ophthalmology. 2025;60(2):e197-e202.	10.1016/j.jcjo.2024.07.018
28	[33]	Chen X, Zhang W, Xu P, et al. FFA-GPT: an automated pipeline for fundus fluorescein angiography interpretation and question-answer. npj Digital Medicine. 2024;7:111.	10.1038/s41746-024-01101-z
29	[34]	Lee GG, Goodman D, Chang TCP. Impact of demographic modifiers on readability of myopia education materials generated by large language models. Clinical Ophthalmology. 2024;18:3591-3604.	10.2147/OPTH.S483024
30	[35]	Chen X, Zhao Z, Zhang W, Xu P, Wu Y, Xu M, et al. EyeGPT for patient inquiries and medical education: development and validation of an ophthalmology large language model. Journal of Medical Internet Research. 2024;26:e60063.	10.2196/60063
31	[36]	Anguita R, Downie C, Ferro Desideri L, Sagoo MS. Assessing large language models' accuracy in providing patient support for choroidal melanoma. Eye (Lond). 2024;38(16):3092-3098.	10.1038/s41433-024-03231-w
32	[37]	Macri CZ, Bacchi S, Wong W, Baranage D, Sivagurunathan PD, Chan WO. A pilot survey of patient perspectives on an artificial intelligence-generated presenter in a patient information video about face-down positioning after vitreoretinal surgery. Ophthalmic Research. 2024;67(1):567-572.	10.1159/000541530
33	[38]	Riazi Esfahani P, Ward J, Yong A, et al. Evaluating ChatGPT's accuracy and readability in responding to common ophthalmology questions. Cureus. 2025;17(7):e87920.	10.7759/cureus.87920
34	[39]	Dal A, Erdağ M, Dikme B, Kutluksaman B. Use of large language models in Turkish information materials for glaucoma patient education: evaluation of readability, accuracy and comprehensiveness. European Eye Research. 2026;6(1):60-69.	10.14744/eer.2025.93723
35	[40]	Yang A, Reid M, Nguyen AM, Nallasamy S, Velez FG, de Alba Campomanes AG, Chang MY. Quality and readability of patient educational materials generated by ChatGPT-4o for pediatric ophthalmologic surgeries. Journal of Pediatric Ophthalmology and Strabismus. 2025;62(6):421-427.	10.3928/01913913-20250404-01
36	[41]	Bondok M, Selvakumar R, Law C, Ing EB, Bakshi NK, Felfeli T. Comparing ophthalmologist and artificial intelligence chatbot responses to patient questions. Clinical Ophthalmology. 2025;19:4293-4300.	10.2147/OPTH.S549820
37	[42]	Lin CW, Lin YC, Chen CY, Wang JJ, Lin HY, Lai IC. Evaluating large language models for glaucoma counseling: a pilot study of ChatGPT and Google Gemini responses in Traditional Chinese. Taiwan Journal of Ophthalmology. 2026;16(1):141-145.	10.4103/tjo.TJO-D-25-00058
38	[43]	Wu LL, Hong AT, Davuluru SS, Moreno-Diaz EA, Gutiérrez-Hernández JC, Martinez-Camarillo JC, et al. Utility of ChatGPT-4o in creating patient handouts in ophthalmology: a comparison with American Academy of Ophthalmology educational materials. Translational Vision Science & Technology. 2026;15(2):14.	10.1167/tvst.15.2.14
39	[44]	Hamzeh N, Lidder AK, et al. Accuracy and readability of Chat Generative Pre-trained Transformer-4 Omni in answering ophthalmology patient questions. Ophthalmology Science. 2026;6(2):101007.	10.1016/j.xops.2025.101007
