# Supplementary material for: Accuracy and Effectiveness of AI-Powered Systems in Patient Counseling, Education, and Management in Optometry and Related Eye-Care Settings: A Systematic Review
Source: Healthcare (Basel). 2026 Jul 24;14(15):2270. doi: 10.3390/healthcare14152270 (PMC13464863; doi:10.3390/healthcare14152270)
Supplement: Supplementary file 1 [file healthcare-14-02270-s001.zip › Supplementary Table S2 JBI 39 Studies FINAL 24 JULY.pdf]

Supplementary Table S2. Study-by-study JBI methodological quality appraisal of the 39 included studies

Appraisal approach: Each included study was appraised using the Joanna Briggs Institute (JBI) checklist most relevant to its design. Because the evidence base included randomized trials, diagnostic/model-validation studies, implementation studies, and AI-output/content evaluations, appraisal findings are summarized narratively rather than converted into a pooled numerical score. The concern level reflects methodological and reporting concerns for interpretation within this systematic review; it does not mean the study was excluded or invalid.

Interpretation of concern levels: Low concern indicates generally clear design, comparator/reference standard, outcome assessment, and reporting. Some concern indicates useful evidence but with limitations such as simulated prompts, limited external validation, limited patient-centered outcomes, or model/prompt dependence. High/unclear concern indicates limited reporting, brief conference/correspondence format, or unclear sampling/evaluation procedures.

No.	Study	DOI / stable identifier	Study design / evidence type	JBI checklist used	Key strengths	Key methodological concerns	Overall appraisal
1	Young et al., 2024 [6]	10.2196/48295	Retrospective case-control / EHR referral-support study	JBI checklist for case-control studies	Real-world electronic health record data; clearly defined referral-support objective; comparator/control structure; clinically relevant referral outcome.	Retrospective design; potential residual confounding and selection bias; performance may depend on the EHR system, referral pathway, and local implementation context.	**Some concern**
2	Dihan et al., 2025 [7]	10.1038/s41433-024-03476-5	Cross-sectional AI-output evaluation of dry eye patient education	JBI checklist for analytical cross-sectional studies	Clear patient-education focus; readability and quality outcomes were relevant to counseling; ophthalmic topic directly aligned with the review scope.	AI outputs were evaluated as generated content rather than real patient encounters; model version, prompt design, and evaluator judgments may limit generalizability.	**Some concern**
3	Wei et al., 2023/2024 [8]	10.1097/j.jcrs.0000000000001288	Brief comparative AI-output evaluation / correspondence	JBI analytical cross-sectional domains, adapted for content evaluation	Directly addresses cataract-surgery patient information; clinically relevant counseling topic.	Very brief report with limited methodological detail; small/unclear sampling frame; limited reporting of evaluator procedures and statistical methods.	**High/unclear concern**
4	Wu et al., 2025 (ChatMyopia) [9]	10.1016/j.isci.2025.113768	AI-agent development and validation for myopia consultation	JBI analytical cross-sectional and diagnostic/model-validation domains	Disease-specific eye-care agent; includes consultation and patient-facing use; evaluates accuracy, safety, and usefulness across text/image-related inquiries.	Model-development and validation setting may not fully represent routine optometry practice; external validation and long-term patient outcomes remain limited.	**Some concern**
5	Yilmaz et al., 2024/2025 [10]	10.1016/j.ijmedinf.2024.105592	Cross-sectional chatbot evaluation for strabismus education	JBI checklist for analytical cross-sectional studies	Patient-education question set; clinically relevant strabismus counseling topic; evaluates chatbot responses using predefined criteria.	Simulated prompts rather than real patient counseling; limited evidence on patient understanding, safety behavior, or follow-up impact.	**Some concern**
6	Wu et al., 2025 (diabetic retinopathy queries) [11]	10.1136/bjo-2024-325861	Cross-sectional LLM response evaluation	JBI checklist for analytical cross-sectional studies	Focuses on diabetic-retinopathy patient questions; clinically relevant outcomes include response quality and appropriateness.	LLM performance may depend on prompt structure and model version; mainly evaluates outputs rather than patient outcomes or clinical workflow impact.	**Some concern**
7	Dawen Wu et al., 2024 [12]	10.1038/s41433-024-03228-5	AI platform validation for strabismus screening and management	JBI diagnostic accuracy / model-validation domains	Clinical strabismus pathway relevance; evaluates AI-supported screening/management rather than text output alone; reports performance in a defined ophthalmic context.	Generalizability depends on population, imaging/clinical workflow, and implementation setting; patient-facing counseling outcomes are indirect.	**Some concern**
8	Armadá-Maresca et al., 2025 [13]	10.3389/fmed.2025.1593556	Clinical performance evaluation of interactive ophthalmology AI platform	JBI analytical cross-sectional / implementation-evaluation domains	Real clinical service context; interactive platform evaluated in ophthalmology; relevant to triage/management support.	Single-center experience; potential selection and implementation bias; limited direct measurement of patient-centered counseling outcomes.	**Some concern**

No.	Study	DOI / stable identifier	Study design / evidence type	JBI checklist used	Key strengths	Key methodological concerns	Overall appraisal
9	Strzalkowski et al., 2024 [14]	10.1186/s40942-024-00579-9	Multicenter expert comparative evaluation of LLM responses	JBI checklist for analytical cross-sectional studies	Multicenter expert comparison; evaluates both accuracy and readability for retinal detachment information; urgent eye-care topic with safety relevance.	Still based on generated responses rather than real patient behavior; model-specific findings may change with AI updates.	**Some concern**
10	Tailor et al., 2024/2025 [15]	10.1001/jamaophthalmol.2025.0351	Randomized trial of AI-generated plain-language summaries	JBI checklist for randomized controlled trials	Randomized design; evaluates interdisciplinary understanding; relevant to simplification of ophthalmology notes and communication.	Outcome focuses on understanding of summaries rather than direct patient counseling; performance may depend on selected note types and AI summary workflow.	**Low concern**
11	Raghu et al., 2023/2024 [16]	10.2147/OPTH.S435052	Comparative diagnostic/risk-assessment evaluation	JBI diagnostic accuracy / analytical cross-sectional domains	Compares AI-generated diabetic retinopathy risk assessment with clinical diagnosis; clinically relevant chronic-disease management topic.	Limited patient-facing counseling evaluation; diagnostic/risk-assessment focus may not directly translate into education or adherence outcomes.	**Some concern**
12	Wolf et al., 2024 [17]	10.1038/s41467-023-44676-z	Randomized controlled trial of autonomous AI for screening and follow-up	JBI checklist for randomized controlled trials	Randomized design; real clinical population; evaluates screening and follow-up uptake in youth; strong relevance to management pathways.	Context-specific implementation; counseling content itself was not the main intervention; longer-term adherence and vision outcomes require further follow-up.	**Low concern**
13	Dow et al., 2023 [18]	10.1016/j.xops.2023.100330	AI-human hybrid workflow evaluation in teleophthalmology	JBI quasi-experimental / cohort domains	Real-world teleophthalmology workflow; evaluates AI-human hybrid detection and management-support pathway.	Non-randomized workflow design; possible site and implementation effects; patient education outcomes not directly measured.	**Some concern**
14	Shruthee et al., 2025 [19]	10.55489/njcm.160920255469	Multicenter prospective diagnostic accuracy study	JBI diagnostic accuracy studies checklist	Prospective multicenter evaluation; cataract detection outcome is clearly defined; relevant to screening linked to patient management.	Primarily diagnostic rather than counseling-focused; applicability to patient-facing education/referral communication is indirect.	**Some concern**
15	Gupta et al., 2023/2024 [20]	10.1097/j.jcrs.0000000000001345	Brief AI-output counseling evaluation / correspondence	JBI analytical cross-sectional domains, adapted for brief reports	Directly focused on cataract-surgery counseling; clinically relevant patient question topic.	Very short report; limited methodological detail; unclear sampling, blinding, and evaluator procedures; no patient outcomes.	**High/unclear concern**
16	Yigen İritaş and İritaş, 2025 [21]	10.18621/eurj.1616435	Cross-sectional ChatGPT patient-education evaluation	JBI checklist for analytical cross-sectional studies	Addresses herpes zoster ophthalmicus education; evaluates AI as patient educator in an ophthalmic condition.	Simulated/content-based evaluation; limited external validation; no direct patient understanding, adherence, or safety behavior outcomes.	**Some concern**
17	Ma et al., 2025 [22]	10.1038/s41746-025-01461-0	Multimodal AI chatbot development and validation	JBI diagnostic accuracy / model-validation domains	Large multimodal ophthalmology AI system; assesses diagnosis and quality of medical responses; relevant to patient inquiries and medical education.	Development/validation setting may differ from routine patient use; patient-centered outcomes and real-world safety monitoring remain limited.	**Some concern**
18	Mathenge et al., 2022 [23]	10.1016/j.xops.2022.100168	Randomized trial of AI assessment and referral uptake	JBI checklist for randomized controlled trials	Randomized design in a low-resource setting; directly evaluates referral service uptake after AI assessment; relevant to management and follow-up.	Counseling content is not isolated as a separate intervention; context-specific barriers may affect transferability.	**Low concern**
19	Mereddy et al., 2024 [24]	10.1109/SCOPES64467.2024.10991151	Conference paper on AI/IoT smart device vision screening	JBI diagnostic/model-validation domains, adapted for conference reporting	Addresses AI-enabled vision screening and diagnosis; relevant to patient-management pathways.	Conference format provides limited methodological and outcome detail; external validation, sampling, and patient-centered outcomes are unclear.	**High/unclear concern**

No.	Study	DOI / stable identifier	Study design / evidence type	JBI checklist used	Key strengths	Key methodological concerns	Overall appraisal
20	Reiter et al., 2025 [25]	10.1016/j.oret.2024.10.029	Prediction/management study in geographic atrophy	JBI cohort / prognostic-prediction domains	Clinically relevant age-related macular degeneration management challenge; compares AI and expert prediction/management support.	Prediction focus rather than direct counseling; generalizability depends on population, imaging data, and expert comparator structure.	**Some concern**
21	Karataş and Durmaz Engin, 2025 [26]	10.1016/j.hlpt.2025.101073	Cross-sectional LLM evaluation for myopia patient queries	JBI checklist for analytical cross-sectional studies	Directly addresses myopia patient questions; evaluates accuracy/comprehensibility dimensions relevant to counseling.	Simulated query evaluation; no direct patient outcomes; model version and prompt choices may limit reproducibility over time.	**Some concern**
22	Dow et al., 2023 [27]	10.2147/OPTH.S422513	Implementation/follow-up evaluation in diabetic retinopathy screening	JBI quasi-experimental / cohort domains	Real clinical screening program; evaluates follow-up after AI-supported screening; relevant to management continuity.	Non-randomized design; local workflow and population may influence outcomes; education/counseling content not separately evaluated.	**Some concern**
23	Al-Sharif et al., 2024 [28]	10.1097/IOP.0000000000002567	Comparative AI versus human-response evaluation	JBI checklist for analytical cross-sectional studies	Patient-query focus in oculoplastics; compares AI and human intelligence; relevant to accuracy of patient-facing responses.	Cross-sectional response evaluation; possible limitations in question selection and evaluator blinding; no patient behavior outcomes.	**Some concern**
24	Kufta et al., 2025 [29]	10.3390/jcm14196968	Readability/content comparison of AI-generated and AAO materials	JBI checklist for analytical cross-sectional studies	Uses professional patient-education comparator; evaluates readability and quality of AI-generated ophthalmology materials.	Content-based evaluation only; no testing with patients; AI output depends on model/prompt/date.	**Some concern**
25	Balas et al., 2025 [30]	10.1016/j.jcjo.2024.11.003	Comparative comprehension study of AI-translated ophthalmic jargon	JBI quasi-experimental / analytical cross-sectional domains	Tests comprehension-related communication outcome; addresses plain-language translation of ophthalmic terminology.	Participant/context details and transferability to routine counseling may be limited; outcomes may depend on selected jargon terms.	**Some concern**
26	Cao et al., 2025 [31]	10.1016/j.aopr.2024.12.002	Randomized controlled study of LLMs in glaucoma patient education	JBI checklist for randomized controlled trials	Randomized comparative design; clinically important chronic-disease education topic; evaluates multiple LLMs.	Patient-outcome follow-up is limited; performance may depend on selected questions, language, and model versions.	**Low concern**
27	Li et al., 2025 [32]	10.1016/j.jcjo.2024.07.018	Cross-sectional evaluation of AI-improved cataract education materials	JBI checklist for analytical cross-sectional studies	Addresses cataract patient education; compares AI-revised materials with online education needs/readability.	Mainly content/readability evaluation; no direct patient testing; prompt/model reproducibility may be limited.	**Some concern**
28	Chen et al., 2024 (FFA-GPT) [33]	10.1038/s41746-024-01101-z	Automated pipeline validation and question-answering study	JBI diagnostic/model-validation domains	Includes report interpretation and question-answering; ophthalmology-specific AI system with relevant patient/clinician communication potential.	Technical and diagnostic components dominate; patient-facing counseling outcomes are indirect; external real-world validation remains limited.	**Some concern**
29	Lee et al., 2024 [34]	10.2147/OPTH.S483024	Cross-sectional readability evaluation of AI-generated myopia materials	JBI checklist for analytical cross-sectional studies	Evaluates demographic modifiers and readability; relevant to equitable patient education and health literacy.	Generated-material study without patient testing; findings depend on prompt conditions and selected demographic modifiers.	**Some concern**
30	Chen et al., 2024 (EyeGPT) [35]	10.2196/60063	Ophthalmology LLM development and validation	JBI diagnostic/model-validation and analytical cross-sectional domains	Ophthalmology-specific LLM; evaluates patient inquiries and medical education; includes development and validation components.	Model-development study may have training/test overlap concerns; real-world patient safety and implementation outcomes remain limited.	**Some concern**
31	Anguita et al., 2024 [36]	10.1038/s41433-024-03231-w	Cross-sectional LLM accuracy evaluation for choroidal melanoma support	JBI checklist for analytical cross-sectional studies	Addresses serious ocular oncology condition; evaluates accuracy of patient-support information with clinical safety relevance.	Likely simulated/content-based prompts; small topic-specific sample; no direct patient outcomes or longitudinal safety monitoring.	**Some concern**

No.	Study	DOI / stable identifier	Study design / evidence type	JBI checklist used	Key strengths	Key methodological concerns	Overall appraisal
32	Macri et al., 2024 [37]	10.1159/000541530	Pilot survey of patient perspectives on AI-generated presenter video	JBI checklist for analytical cross-sectional studies	Includes patient perspectives; evaluates acceptability of AI-generated patient-information video content.	Pilot design; likely limited sample size and generalizability; evaluates perceptions more than clinical understanding or behavioral outcomes.	**Some concern**
33	Riazi Esfahani et al., 2025 [38]	10.7759/cureus.87920	Cross-sectional ChatGPT response-quality and readability evaluation	JBI checklist for analytical cross-sectional studies	Uses Google Trends-derived patient questions; two board-certified ophthalmologists assessed responses; applies multiple quality/readability tools.	Only 22 questions; one ChatGPT model/session approach; no real patient testing; glaucoma/cataract responses showed weaker appropriateness.	**Some concern**
34	Dal et al., 2026 [39]	10.14744/eer.2025.93723	Cross-sectional multilingual LLM patient-education evaluation	JBI checklist for analytical cross-sectional studies	Evaluates Turkish glaucoma education; compares multiple LLMs with a professional brochure; ophthalmologists assessed accuracy/comprehensiveness.	Simulated brochure-question format; limited to one language and disease area; patient comprehension or behavior not directly tested.	**Some concern**
35	Yang et al., 2025 [40]	10.3928/01913913-20250404-01	Comparative evaluation of ChatGPT-4o pediatric surgery PEMs	JBI checklist for analytical cross-sectional studies	Compares AI-generated materials with AAPOS resources; evaluates English and Spanish outputs; uses defined quality and readability measures.	Content-based study without patient testing; limited number of procedures; model update effects may affect reproducibility.	**Some concern**
36	Bondok et al., 2025 [41]	10.2147/OPTH.S549820	Retrospective cross-sectional comparison of chatbot and ophthalmologist answers	JBI checklist for analytical cross-sectional studies	Large random sample of AAO forum questions; real patient questions; board-certified ophthalmologist comparator and rated accuracy/readability/empathy.	Retrospective public-forum data; limited clinical context; no direct measurement of patient understanding or outcomes.	**Low concern**
37	Lin et al., 2026 [42]	10.4103/tjo.TJO-D-25-00058	Pilot cross-sectional LLM evaluation in Traditional Chinese	JBI checklist for analytical cross-sectional studies	Non-English glaucoma counseling focus; 20 frequently asked questions; three ophthalmologists independently rated appropriateness.	Pilot scale; simulated translated questions; limited domains and no patient comprehension or behavior outcomes.	**Some concern**
38	Wu et al., 2026 [43]	10.1167/tvst.15.2.14	Comparative bilingual handout-quality evaluation	JBI checklist for analytical cross-sectional studies	Compares ChatGPT-4o with AAO handouts in English and Spanish; uses multiple readability metrics and three ophthalmologist graders per language.	Content-based handout evaluation rather than patient trial; selected AAO topics and prompt conditions may limit generalizability.	**Low concern**
39	Hamzeh et al., 2026 [44]	10.1016/j.xops.2025.101007	Retrospective cross-sectional evaluation of real MyChart patient questions	JBI checklist for analytical cross-sectional studies	Uses real patient-submitted questions; two independent ophthalmologist reviewers plus adjudication; evaluates accuracy/completeness and readability.	Single-institution retrospective sample; convenience stopping rule; patient understanding and clinical outcomes were not directly measured.	**Low concern**

Appraisal color key: pale green = low concern; pale amber = some concern; pale red = high/unclear concern. The written appraisal category remains the primary interpretation.

Abbreviations: AI, artificial intelligence; EHR, electronic health record; JBI, Joanna Briggs Institute; LLM, large language model; PEM, patient education material. The appraisal table is intended to support narrative synthesis and transparency; it is not a meta-analytic risk-of-bias score.
